# Does engagement predict research use? An analysis of The Conversation Annual Survey 2016

**DOI:** 10.1371/journal.pone.0192290

**Published:** 2018-02-07

**Authors:** Pauline Zardo, Adrian G. Barnett, Nicolas Suzor, Tim Cahill

**Affiliations:** 1 Faculty of Law, Queensland University of Technology, Brisbane, Queensland, Australia; 2 Institute for Health and Biomedical Innovation, Queensland University of Technology, Brisbane, Queensland, Australia; 3 KPMG, Melbourne, Australia; University of Sheffield, UNITED KINGDOM

## Abstract

The impact of research on the world beyond academia has increasingly become an area of focus in research performance assessments internationally. Impact assessment is expected to incentivise researchers to increase engagement with industry, government and the public more broadly. Increased engagement is in turn expected to increase translation of research so decision-makers can use research to inform development of policies, programs, practices, processes, products, and other mechanisms, through which impact can be realised. However, research has shown that various factors affect research use, and evidence on ‘what works’ to increase decision-makers’ use of research is limited. *The Conversation* is an open access research communication platform, published under Creative Commons licence, which translates research into news articles to engage a general audience, aiming to improve understanding of current issues and complex social problems. To identify factors that predict use of academic research and expertise reported in *The Conversation*, regression analyses were performed using *The Conversation* Australia 2016 Annual Survey data. A broad range of factors predicted use, with engagement actions being the most common. Interestingly, different types of engagement actions predicted different types of use. This suggests that to achieve impact through increased engagement, a deeper understanding of how and why different engagement actions elicit different types of use is needed. Findings also indicate *The Conversation* is overcoming some of the most commonly identified barriers to the use of research: access, relevance, actionable outcomes, and timeliness. As such, *The Conversation* offers an effective model for providing access to and communicating research in a way that enables use, a necessary precursor to achieving research impact.

## Introduction

In 2018, for the first time, Australian universities will be assessed on 1) the extent to which they engage with industry, government and other research end-users, and 2) the impact that their research has had on the world beyond academia [1]. The Australian Engagement and Impact Assessment runs in parallel to the established Excellence in Research Australia (ERA) exercise, focused on research funding, publications, citations and peer review [1]. While impact assessment has been on and off the Australian Government agenda for some time, it was the United Kingdom that implemented the world’s first national impact assessment in 2014 [2,3]. The UK Research Excellence Framework (REF) included an impact assessment based on detailed case studies of the impacts research has had beyond academia that could be traced to an underpinning research base [4].

The UK’s action on research impact assessment spurred further Australian developments. In 2015 the Academy of Technology, Science and Engineering (ATSE) ran the Research Engagement Australia (REA) pilot. The REA pilot developed and tested income-based metrics to demonstrate engagement between academia and industry. Focusing on engagement metrics addressed two key criticisms of the UK’s case study approach to impact assessment [5]. First, metrics that use existing data sets minimise burden on the academic sector in regards to collecting evidence of impact [6,7]. Secondly, while impact can take a long time to be achieved and is not in the direct control of academics [3], engagement metrics based on industry-funded research demonstrate vested relationships with industry, and can indicate potential future impact. In 2015, the Australian Government also commissioned a review of research funding which recommended that assessment of research impact be added to national research performance assessments [8].

In December 2015 the Australian Government launched the National Innovation and Science Agenda (NISA) that announced the introduction of a research impact assessment [9]. The purpose of the impact assessment, as outlined in the NISA, was to incentivise higher education institutions to increase engagement with industry and government to increase the positive impacts that research has on the economy and society more broadly. The central hypothesis of this approach is that increased engagement drives increased impact. This premise is supported by research that has identified close collaboration, increased interaction as well as access, trust, relevance and communication as key factors that affect research use by decision-makers in industry and government [10–14].

The problem with focusing solely on engagement as an indicator of future impact is the failure to acknowledge the significant challenge of getting decision-makers to use research evidence. To get from engagement to impact, research must be used in some way. Research must become an input to the decision-making, development, production, implementation or use of policies, programs, practices, products, services, etc., the mechanisms that deliver impact beyond academia. Research use has been defined as the application of research evidence and expertise to inform decision-making within planning, development, implementation and evaluation processes [15–19]. Studies of government decision-makers use of research have shown that different types of research evidence are used. Decision-makers directly commission research relevant to an issue they are facing, search for existing individual studies or literature review or systematic reviews of research, and decision-makers also seek out expert evidence-based advice directly from academics who are recognised experts in their field [16,20–24].

There are also different ways in which research evidence is used. Types of research use have commonly been categorised as: instrumental use, which refers to direct application of research-based evidence; conceptual use, referring to use of research evidence to inform understanding, discussion and/or debate without direct application; and tactical, political or symbolic use, which refers to the use of research evidence to inform a decision or position previously established [10,13,15,25–28].

The definitions and types of use outlined above align with the Australian Research Council definition of impact, which built on the UK’s definition of research impact [3]: ‘The contribution that research makes to the economy, society, environment and culture beyond the contribution to academic research’. This aligns with the academic definitions and types of use outlined above. It suggests that research must be used in some way that contributes to the world beyond academia, but does not specify a specific type of use or contribution. Impact case study templates used in both the UK and Australia require researchers to describe how their research has been used outside of academia how that use represents an important contribution [2, 23].

Engagement has been clearly differentiated from use in the work of Redman et al. [15,29,30], who define engagement as actions ‘that create a bridge between the potential reflected in the capacity to use research and the eventual outcome of research application’ [15]. The definition of engagement adopted for the Australia research impact assessment is ‘The interaction between researchers and research end-users outside of academia, for the mutually beneficial transfer of knowledge, technologies, methods or resources’ [31]. Here, engagement refers to how research-based outputs are transferred to, accessed and shared between researchers and users of research outside of academia. This aligns to Redman et al [15] identification of research access, research appraisal and interactions between decision-makers and researchers as engagement actions.

The 2017 Innovation and Science Australia Performance Review found there are limited mechanisms to support research translation and collaboration between researchers and industry [32]. Research translation and implementation science are fields of research that have developed in response to the substantial challenges faced by those who have attempted to increase use of research in industry and government [33,34]. There is an extensive research base on the factors affecting decision-maker research use [10,11,35–40]. There has also been extensive work in using identified factors and relevant theories and frameworks [41–44] to design interventions aimed at increasing use of research [12,14,18,41–45]. Many of these frameworks are focused on: increasing interaction and collaboration between researchers and decision-makers, for example through co-production [41–44] of research and implementation projects; increasing access to and accessibility of research, e.g. through synthesising research and improving research communication; and also building capacity for decision-makers to engage with research evidence and researchers and vice versa, often though training and facilitation [12,14].

While it has been established that many factors can affect research use and many interventions developed, high quality empirical research testing interventions that aim to increase decision-makers’ research use is more limited [12,14,29,45]. A recent systematic review of research covering the years 1990 to 2015 identified just 16 studies that specifically sought to test interventions aimed at increasing decision-makers’ use of research [12]. Seven of these interventions effectively increased research use, the other nine failed to show any change in behaviour. The authors concluded that ‘skill or the intention to use evidence, in itself, cannot be regarded as a reliable indicator of behaviour change in practice’. A complex interplay of factors can affect research use, which can make the transition from engagement to research use and subsequent impact extremely difficult. Engagement is only a first, albeit necessary, step on a challenging pathway to impact that has not yet been extensively mapped, with no definitive means to effectively navigate.

The systematic review by Langer (2016) [12] showed that interventions with the most reliable evidence of increasing decision-makers’ research use are those that facilitated research access and communication, and built skills for research use [12]. This is supported by the recent review by Sarkies et al. (2017) [14] which identified three studies with experimental designs that tested interventions. Two of the studies were focused on access and communication. The other focused on use of knowledge brokering to support decision-makers to use research. Access mechanisms used in these effective interventions were mainly online evidence portals designed for specific research fields and systematic or other literature reviews that summarised evidence for decision-makers. Targeted and tailored research messages were the most effective communication approaches. While there are many factors affecting research use that are not directly in the control of academia, research access and communication are factors that academics and research institutions can increase and improve.

There has also been a great deal of work on increasing access to research through the open access publishing movement, however there are a limited number of studies that have examined the use of open access academic research by those outside of academia [46,47]. A recent review found just 53 studies published between 2001 and mid 2017 on use of open access research by those outside of academia [46]. The two largest and most comprehensive of these studies were on use of institutional research repositories [48]. These studies showed that there are people outside of academia that seek out and use research across a range of fields for both work-related and personal reasons [46–48].

It is clear from the Engagement and Impact Assessment in Australia and much research in the field of research translation that direct engagement, interaction, collaboration and co-production of research are needed to support increased use and impact of research [12,14,15,31,41,49,50]. For example, the engagement indicators used in the the Engagement and Impact Assessment in Australia are based on establishment of research partnerships, and collaboration and co-production require researchers and decision-makers or end-users to directly work together. The evidence that people are accessing and using research outside of direct engagement and translation efforts [46–48], coupled with the consistent finding that access and accessibility are critical factors enabling research use [11,12], highlights the need to better understand independent research use enabled by open access platforms and identify whether this type of use also has a relationship with indirect engagement actions.

### The Conversation

*The Conversation* is an online research communication platform that provides access to academic research evidence that has been ‘translated’ and communicated in a form more accessible to general audiences than traditional academic journal articles. *The Conversation* articles include papers that report on or review research findings as well as articles that offer academic expert opinion and analysis on current news issues. *The Conversation* was established in 2011, with the funding from four Australian universities and one Australian government funded research institution. *The Conversation* sought to provide better understanding of current and complex issues in the news through high quality, independent journalism based on research evidence and academic expertise. *The Conversation* has a strong open access mandate and ethic, assuring their content will remain free to both read and share [51].

*The Conversation* has experienced rapid uptake since its launch in 2011. Chapters have since been established in the US, UK, South Africa and France. The Conversation attracts a monthly readership of approximately 3.8 million with a further 35 million readers reached each month through republication [51]. *The Conversation* publishes short journalistic style articles written by academic researchers, who must have doctoral research qualifications or be studying for doctorate. *The Conversation*’s editors, who have journalism backgrounds and expertise, select articles for publication and guide development of article content to ensure it is relevant and accessible to a general audience. *The Conversation* articles are published using a Creative Commons licence, which means other organisations and individuals can access them for free and can also republish them in full at no cost [51].

Each year *The Conversation* undertakes an annual survey of their Australian readership. The survey includes a broad range of questions including reader demographics and questions relating to readers’ main purpose for reading *The Conversation*, what they value about *The Conversation*, and how frequently they read *The Conversation*. Importantly, the survey also asks about the actions they take after reading articles on *The Conversation* and whether and how readers have used *The Conversation* articles to inform decision-making related to their work and their lives. The actions outlined in the survey are focused on actions that involve engagement with *The Conversation* content, including: ‘left a comment on the article’, ‘shared an article on social networks’, ‘discussed with friends or colleagues’, ‘contacted a local politician or government official’. The survey also asked about types of use of articles including: to inform development of policy, or to inform personal attitude or behaviour change. The types of engagement action and use of research captured in the survey are much broader than alternative metrics or ‘altmetrics’ often used as proxy measures of engagement and impact [52–54]. *The Conversation* annual survey therefore provides a unique opportunity to explore how the broad range of factors examined in the survey, including engagement, affect the use of research read in *The Conversation*.

This paper reports the findings of regression analyses undertaken on the data collected via *The Conversation* Australia Annual Survey 2016. The analysis aimed to answer the following research questions:

- Do engagement actions predict use of information read in *The Conversation* articles?
- What other factors predict use of information read in *The Conversation* articles?

## Materials and methods

*The Conversation* developed *The Conversation* Annual Survey 2016, with input from Dr Zardo. Dr Zardo led inclusion of questions on use of research that forms the focus of this study (see S1 File). The survey was made available to readers via *The Conversation* Australian website and through their online newsletter mailed to subscribers. It was also advertised via *The Conversation* social media. The survey was open from 7 to 15 April 2017. Survey Monkey was used to distribute the survey and collect responses. The method of survey collection was non-random and we cannot calculate a survey response rate or compare the characteristics of respondents and non-respondents.

A low risk human research ethics application was approved by the Queensland University of Technology University Human Research Ethics Committee to analyse the data. The first survey question asked consent for the data to be used in subsequent reporting. Eighteen people (0.23% of total sample) did not give consent, leaving a total sample for analysis of 7,772 consenting participants for analyses.

Data on use of research and academic expertise reported in *The Conversation* articles were generated from the survey question ‘Have you used articles from *The Conversation* to do any of the following?’ with the following response options, each asking about a different type of use (tick as many as apply):

- Develop strategy, policy, presentations, decisions and/or directions which have been documented, for example, in policy briefs, papers, projects plans or reports, PowerPoints, etc.
- Further support existing an strategy, policy, program or business decisions
- Inform general understanding, discussion and debate on strategy, policy, project or business topics
- Change my own behaviour and/or attitudes in my personal life

These are the four measures of use that we aimed to predict. The first three types of use align to the instrumental, tactical/symbolic and conceptual types of research use discussed in the introduction and are specifically related to use of articles in relation to work-related decision-making. The fourth seeks to identify use of research to inform one’s personal decision-making.

Data on engagement actions were generated from the question ‘What actions have you taken as a result of reading an article on The Conversation?’ with the following response options (tick as many as apply):

- Republished the article
- Left a comment on the article
- Shared an article on social networks (e.g. Facebook, Twitter) or by email
- Discussed with friends or colleagues
- Printed to read or share
- Contacted the author to discuss their ideas
- Contacted the author to work with them
- Contacted the author to ask about studying with them or at their university
- Used the article in a report
- Used the article as a classroom resource or as basis of discussion with students
- Contacted a local politician or government official
- Undertaken further research
- None

The actions listed here represent dissemination, communication and other activities undertaken by the reader to engage others or the author, or engage with*The Conversation* content, or led to engagement with other content. Each response to these questions was automatically made into an individual variable with a binary (yes/no) outcome.

The survey also asked about: age, gender, state of residence, place of residence (city, regional, rural), education, income, sector worked within; employment position; business owner and business type.

### Statistical methods

To determine the factors that predicted the four types of use of *The Conversation* articles outlined above, two methods of regression analysis were used: logistic regression and classification trees. Regression methods were selected because our main aim was to find which variables predicted use. As there is current limited published quantitative evidence of factors that predict research use, an inclusive approach was used and each survey question, excluding the question on research use, which is the outcome, and the question on university affiliation, was treated as a potential predictor of research use. See S2 File for full list of included predictor variables. We used two regression methods as we aimed to see if there was convergence or divergence in the predictors selected. This approach was taken, as there is often no single regression method that can be definitively described as the ‘best’ method. See the following references for further examples of studies using of a mix of regression methods [55–57].

The logistic regression analysis was undertaken in steps. To ensure that the predictor variables were not highly correlated with each other, variance inflation factors (VIFs) were used and were less than 5 for all variables, and were between 1 and 1.3 for variables that significantly predicted each type of use, indicating that collinearity was not an issue. The first step in the logistic regression was a univariate analysis where each variable was independently tested against research use. Then in the second step, all factors that were significant predictors of research use were entered into a regression model in a single block. Then all significant variables in the first block were entered into a second model in one block. This process was repeated until all factors in the block made a statistically significant contribution to the model. See S3 File for details of logistic regression results and classification tree results.

A classification tree was generated for each of the four types of research use. Classification trees use predictors to split the data into the two most distinct groups, e.g. in a dataset of cause of death, deaths from lung cancer (yes/no) might be split by smoking status (yes/no). Our dependent variable was research use and we included all other survey question responses as potential predictors. After each split is made the two nodes created are considered for further splitting until no further splits can be made. The stopping rule for splits is based on the improvement in model fit, so each split must improve the model’s predictive ability.

Each time a split is made every predictor is considered using every possible grouping or cut-off, and the variable that creates the biggest difference in the dependent variable is chosen. This intensive selection makes classification trees vulnerable to over-fitting and hence 10-fold cross-validation is used to avoid selecting spurious predictors. A key advantage of classification trees is that they are able to consider multiple potential predictors but generally avoid the problem of multicollinearity because the staged approached makes it unlikely that two highly correlated predictors will be selected. Trees are also very useful at uncovering interactions between predictors. Trees are generally inefficient for modelling linear or non-linear associations with continuous predictors; however most of our predictors were categorical.

Probability ratios for each node of each tree were calculated in relation to the root node, where the root node is the overall sample before any splits are made. For example, if the probability of research use in the root node was 0.1 and the probability of use in women was 0.2 then the probability ratio for women would be 2.

## Results

Overall results of the regression analyses are presented in Table 1 below. The results of the logistic regression are discussed together with the results of the classification tree below. Please note this table only includes predictors that showed a realtionships to the type of use outcome.

**Table 1:** Predictors of use of a *The Conversation* article. Positive predictors are in green and negative in orange.

	Logistic Regression—odds ratio (p value)				Classification Tree—probability ratio			
Engagement Actions	**Inform**	**Support**	**Discuss**	**Personal**	**Inform**	**Support**	**Discuss**	**Personal**
Republished the article	**1.93 (>0.001)**	**1.78 (>0.001)**	**2.04 (>0.001)**		**4.06**			
Left a comment on the article	**1.29 (0.005)**							
Shared an article on social networks (e.g. Facebook, Twitter) or by email	**1.25 (0.008)**	**1.27 (0.002)**						
Discussed with friends or colleagues		**1.62 (>0.001)**		**2.01 (>0.001)**		**3.47**	**1.06**	**1.13**
Printed to read or share	**1.53 (>0.001)**	**1.52 (>0.001)**						
Contacted the author to discuss their ideas		**1.49 (>0.001)**	**1.65 (>0.001)**					
Used the article in a report	**4.29 (>0.001)**	**2.93 (>0.001)**	**2.47**		**3.13**	**2.47**		
Used the article as a classroom resource or as basis of discussion with students	**1.53 (>0.001)**	**1.23 (0.046)**						
Contacted a local politician or government official	**1.71 (>0.001)**	**1.83 (>0.001)**						
Undertaken further research	**1.84 (>0.001)**	**1.73 (>0.001)**	**2.03 (>0.001)**	**1.42 (>0.001)**	**4.53**			
None							**0.83**	
Main Reasons for Reading TC	**Inform**	**Support**	**Discuss**	**Personal**	**Inform**	**Support**	**Discuss**	**Personal**
To explain the news			**1.35 (>0.001)**	**1.48 (>0.001)**				**1.24**
To assist in my work / research	**2.48 (0.000)**		**1.45 (>0.001)**	**0.64 (>0.001)**	**3.73**		**1.27**	
To explore issues I care about / for interest			**1.75 (>0.001)**	**1.40 (>0.001)**				
For expert opinions and facts				**1.47 (>0.001)**			**0.93**	**1.05**
To read about issues not covered elsewhere				**1.25 (>0.001)**				
To find out about new research and breakthroughs				**1.25 (>0.001)**				
It is better than the alternatives				**1.38 (>0.001)**				
Value	**Inform**	**Support**	**Discuss**	**Personal**	**Inform**	**Support**	**Discuss**	**Personal**
Academic expertise				**1.14 (0.001)**				
Creative commons / open source publishing				**0.90 (0.001)**				
Author disclosures				**1.13 (>0.001)**				
Opportunity to engage with people outside my normal networks		**1.10 (>0.001)**						
Publication Follow Up	**Inform**	**Support**	**Discuss**	**Personal**	**Inform**	**Support**	**Discuss**	**Personal**
Invitations to speak at conferences	**4.80 (>0.001)**							
Dashboard Use	**Inform**	**Support**	**Discuss**	**Personal**	**Inform**	**Support**	**Discuss**	**Personal**
Demonstrating engagement to apply for research funding		**2.52 (0.024)**						
Other (please specify)	**7.25 (0.022)**							
Employ Status	**Inform**	**Support**	**Discuss**	**Personal**	**Inform**	**Support**	**Discuss**	**Personal**
Employed, full time		**1.78 (>0.001)**	**1.91 (>0.001)**	**0.77 (0.001)**				
Employed, part time				**0.84 (0.037)**				
Unpaid work / volunteer		**1.49 (0.005)**						
Retired	**0.69 (0.001)**							
Income	**Inform**	**Support**	**Discuss**	**Personal**	**Inform**	**Support**	**Discuss**	**Personal**
$49,999 to $99,999		**1.39 (0.010)**						
$100,000 -$149,000	**1.47 (0.009)**	**1.62 (>0.001)**						
$150,000 -$299,000	**1.78 (>0.001)**	**1.56 (0.001)**						
$300,000 plus	**2.33 (>0.001)**	**1.51 (0.034)**						
Prefer not to say								
Education	**Inform**	**Support**	**Discuss**	**Personal**	**Inform**	**Support**	**Discuss**	**Personal**
Undergraduate Degree				**0.82 (0.040)**				
Graduate/Postgraduate Certificate				**0.81 (0.038)**				
Graduate/Postgraduate Diploma								
Master's Degree	**1.77 (>0.001)**			**0.61 (>0.001)**				
Doctorate								
Sector	**Inform**	**Support**	**Discuss**	**Personal**	**Inform**	**Support**	**Discuss**	**Personal**
Consulting & Strategy		**2.03 (>0.001)**						
Energy & Resources		**1.62 (0.045)**						
Government, Policy or Public Sector		**1.42 (0.017)**						
Media / Journalism		**0.24 (>0.001)**						
Role title	**Inform**	**Support**	**Discuss**	**Personal**	**Inform**	**Support**	**Discuss**	**Personal**
Chairperson, director, CEO/CFO,COO, owner, partner or proprietor	**1.82 (0.001)**	**1.91 (>0.001)**		**0.75 (0.016)**		**3.29**		
General manager, department head, senior executive, manager, or professional	**1.71 (0.001)**	**1.47 (0.006)**						
Politician, policy officer, or government employee	**3.22 (>0.001)**	**1.57 (0.012)**			**5.53**			
Media professional (e.g., journalist, writer, broadcaster, advertiser, PR)				**0.69 (0.031)**				
Not applicable				**0.71 (0008)**				
Business Type	**Inform**	**Support**	**Discuss**	**Personal**	**Inform**	**Support**	**Discuss**	**Personal**
Construction				**2.42 (0.004)**				
Accommodation, cafes and restaurants	**0.27 (0.020)**							
Transport and storage	**4.16 (0.014)**			**3.94 (0.021)**				
Finance and insurance				**1.92 (0.041)**				
Health and community services				**1.80 (0.001)**				
Cultural and recreational services				**1.65 (0.033)**				
Age	**Inform**	**Support**	**Discuss**	**Personal**	**Inform**	**Support**	**Discuss**	**Personal**
35–49				**0.78 (0.010)**				
50–64				**0.55 (>0.001)**				
65 or older				**0.44 (>0.001)**				

The total sample of (7772) had an overall probability of:

- 15% for using *The Conversation* articles to inform development of strategy, policy, programs, etc.
- 17% for using *The Conversation* articles to support work-related decisions previously made.
- 71% probability of using *The Conversation* articles to inform work-related discussion and debate.
- 53% of using information read in *The Conversation* articles to inform personal attitude or behaviour change.

### Use of research to inform work-related discussion, debate

‘To assist with work’ was the only factor that predicted this type of use across both regression analyses. The tree showed a 27% increase in probability and the logistic regression showed 1.45 greater odds of this type of use. The logistic regression identified a greater number of predictors than the tree. The engagement predictors identified showed ‘used the article in report’, ‘undertake further research’ and ‘republish the article’ had more than twice the odds of this type of use compared to those who didn’t engage in these actions.

### Use of research to develop strategy, policy, presentations, decisions and/or directions, etc.

‘Used the article in a report’ was the first split in the classification tree for this type of use and showed that the 14% of the respondents who had used a *The Conversation* article in a report has 3.1 times greater probability of using an article in this way. The logistic regression indicated those who used an article in a report had a 4.3 times greater odds of this type of use.

Logistic regression also indicated that ‘politician, policy officer and other government worker’ (referred to from here on in as policy decision-makers) had 3.2 times higher odds of this type of use compared to participants in other roles. The classification tree showed that the 6% (N = 466) of respondents who used an article in a report, identified ‘assist with work’ as a main reason for reading *The Conversation*, and respondents who had worked as a policy decision-maker were 5.5 times more likely to use an article in this way. This combination of factors was the strongest predictor of this type of use in the classification tree.

The logistic regression identified ‘Chairperson, Director, Chief Executive Officer, Chief Financial Officer, Chief Operating Officer, Owner, Partner or Proprietor’ and ‘General Manager, Department Head, Senior Executive, Manager, or Professional’ as predictors of this type of use, however the classification tree did not distinguish between these and other positions (other than policy decision-makers).

The classification tree also showed that, excluding policy decision makers, the 4% of participants who ‘used an article in a report’, indicated ‘assist with work’ as a main reason for reading *The Conversation*, and indicated they undertook ‘further research’, had a 4.5 times greater probability of this type of use compared with the sample average.

‘Assist with work’ was the basis of the second split in the classification tree on this type of use. The tree showed that the 8% (N = 622) of those who had ‘used the article in a report’, and indicated ‘to assist with work’ as a main reason for reading *The Conversation*, were 3.7 times more likely to use an article in this way. Both regression analyses indicated similar results in regards to ‘assist with work’, with an odds ratio of 1.45 in the logistic regression, and a probability ratio of 1.27 in the tree.

### Use of research to support a work-related decision or action that had already been made

‘Used the article in a report’ predicted this type of use in the logistic regression. ‘Used article in a report’ was also the first split in the classification tree for this type of use. The tree showed 14% of those who used a *The Conversation* article in a report were 2.5 times more likely to use an article in this way, compared to the sample average.

Employment as ‘Chairperson, Director, Chief Executive Officer, Chief Financial Officer, Chief Operating Officer, Owner, Partner or Proprietor’ and ‘General Manager, Department Head, Senior Executive, Manager, or Professional’ as well as a policy-decision-makers, predicted this type of use across both regression methods. The tree showed that those who ‘used an article in report’ and were employed in one of these three employment positions were 3.3 times more likely to use research in this way compared with the sample average.

The logistic regression indicated that the ‘Chairperson, Director, Chief Executive Officer, etc.’ group had nearly twice the odds of using research in this way, compared to those in other employment positions. At 1.91, this was the highest odds ratio of the predictive employment positions. The logistic regression also showed that policy decision-makers and ‘General Manager, Department Head, Senior Executive, etc.,’ had respectively 1.57 and 1.47 greater odds to use research in this way compared with other employment positions.

The logistic regression also showed that those who ‘discussed research with friends and colleagues’ had 1.62 greater odds of this type of use compared to those who did not indicate that they discussed articles. The classification tree showed that the 5% (N = 389) who used an article in a report, were employed in senior roles or were policy decision makers and discussed articles with friends and colleagues were 3.5 times more likely to use research in this way. Therefore, for those in policy and senior roles, discussing articles with friends and colleagues further increased their likelihood of using articles in this way.

### Use of research to inform personal attitude and behaviour change

‘Discussed with friends and colleagues’ was the first split on the tree for this type of use. The tree showed that the 80% of participants who discussed articles with friends and colleagues’ were 1.13 times more likely to use articles in this way compared to the sample average. The classification tree showed that the 48% of participants who ‘discussed articles with friends and colleagues’ and also read The *Conversation* ‘to explain the news’ were 1.24 times more likely to use articles in this way.

The logistic regression found that those ‘who discussed an articles with friends and colleagues’ had twice the odds of this type of use, compared to those who did not indicate they discussed articles. Participants who indicated they read *The Conversation* ‘to explain the news’ had 1.48 times greater odds of using an article in this way, compared to those that did not indicate this main reason.

The logistic regression showed participants who indicated ‘expert opinion and facts’ as a main reason for reading *The Conversation* had 1.47 greater odds of using an article in this way. The classification tree showed a more complicated picture. The 22% of participants who discussed *The Conversation* articles with friends and colleagues, but did not indicate ‘explain the news’ as main reason for reading *The Conversation*, and instead indicated ‘for expert opinion and facts’ as their main reason were only 1.05 times more likely to use research in this way, compared to the sample average.

## Discussion

Analysis of *The Conversation* 2016 Annual survey has yielded novel insights on how information in *The Conversation* articles is being used to inform readers’ decision-making in their work and personal lives. This study makes an important contribution to the high quality quantitative literature on factors that predict decision-maker use of academic research evidence and expertise [11,16,58]. In particular this study draws on data from a broad range of sectors and included clearly defined types of use of *The Conversation* articles addressing issues identified as challenges to the interpretation of outcomes in previous research [11,12]. Further, this study has included a focus on use of *The Conversation* articles in personal decision-making and shown that the factors that predict individuals’ use of *The Conversation* articles in personal decision-making differ from the factors that influence work-related decision-making.

This study set out to answer two questions: do engagement factors predict use?; and what other factors predict use?. The key factors that predicted work-related use of *The Conversation* articles were related to engagement actions taken after reading an article, being employed in senior-level and policy-related employment positions. Work related use showed much higher growth in predictive capability with additional factors, suggesting it is more dependent on a number of specific factors working in cooperation. This aligns with previous research focused in health policy contexts, which found that the predictive power of factors found to affect research use improved when factors were ‘manipulated simultaneously’ [59]. Of the three key factors that affected personal decision-making regarding attitude and behaviour change, two were related to participants’ main reasons for reading *The Conversation*, and one was an engagement action. Importantly, this study demonstrates the potential for end-user surveys to form part of the suite of metrics seeking to demonstrate research engagement and impact as part of national research performance assessments [3].

### Engagement actions predict use

Engagement actions were the most common predictors of article use. Engagement actions predicted all four types of article use, across both regression models. Employment position was the only other variable that also predicted all four types of use, but not as frequently as engagement actions did, and only in the logistic regression. Engagement actions more frequently predicted work-related use of *The Conversation* articles, compared with use of *The Conversation* to inform personal attitude and behaviour change. Interestingly different engagement actions predicted different types of use. For example ‘discussed with friends or colleagues’ predicted use of an article to further support a work-related decision that had been previously made and also predicted use of an article to inform the reader’s own personal attitude or behaviour change, but did not predict use of an article to directly inform development of strategy, policy, etc. These findings support the hypothesis that increased engagement can support increased research impact, but also highlight that not all engagement actions have the same effect. This suggests that a deeper understanding of how different engagement actions elicit different types of research use is needed to achieve research impact through increased engagement [12,53,60–62].

### Key predictors of research use

The advantage of using both the logistic regression and classification tree analyses is that logistic regression identifies factors that independently predict use and the classification tree highlights relationships between factors that predict use. This is important as it has been highlighted that the lack of identification of relationships between variables is a limitation of regression analyses [36]. As Table 1 demonstrates, a broad range of factors significantly predicted the each type of article use across the two methods. For sake of brevity, the remainder of the discussion will focus on factors identified as statistically significant predictors across both logistic regression and classification tree analyses. Table 2 provides an overview of the factors that predicted each type of research use across both regression methods.

**Table 2:** Overview of key factors predicting use of *The Conversation* articles.

Type of use of *The Conversation* articles	Factors predicting use in both logistic regression and classification tree	Related survey question
Inform development of strategy, policy, programs, etc.	Used article in a report	What actions have you taken as a result of reading a *The Conversation* article? (engagement actions)
	Republished the article	
	Undertaken further research	
	To assist in my work/research	What are the main reasons you read *The Conversation*?
	Politician, policy officer, or government employee	What is your role title?
Support an existing strategy, policy, program, etc., decision.	Used article in a report	What actions have you taken as a result of reading a *The Conversation* article? (engagement actions)
	Discussed with friends or colleagues	
	Chairperson, director, CEO/CFO, COO, owner, partner or proprietor	What is your role title?
	General manager, department head, senior executive, manager, or professional	
	Politician, policy officer, or government employee	
Inform understanding, discussion and debate related to work	To assist in my work/research	What are the main reasons you read *The Conversation*?
Inform own attitude or behaviour change in personal life	Discuss with friends or colleagues	What actions have you taken as a result of reading a *The Conversation* article? (engagement actions)
	To explain the news	What are the main reasons you read The Conversation
	For expert opinion and facts	

#### Instrumental use of *The Conversation* articles

‘Used the article in a report’ was the strongest predictor of use of *The Conversation* articles to develop strategy, policy, presentations, decisions and/or directions, etc. across both regression methods. Using research to directly inform development of policy, program, practice and other action has been described as ‘instrumental’ research use [10,13,15,25]. Importantly, this finding indicates that *The Conversation* articles have been relevant to decision-makers needs and actionable within their work-related context. This means that *The Conversation* is overcoming some of the most commonly identified barriers to decision-maker use of research: access, relevance, actionable outcomes, and timeliness [11,40,63].

Finding that use of an article in a report predicts use of an article to inform development of policy, strategy, project etc., makes intuitive sense, as these are both direct, instrumental uses of research. In testing assumptions for inclusion of predictors, ‘used article in a report’ was included because it was correlated with the outcome variable but was not highly correlated to other predictors which is a necessary assumption for effective logistic regression analysis [64]. Further, the outcome ‘using article to inform policy, strategy, program, project documents, etc.’ includes a number of different outputs, therefore it is possible that those who have used *The Conversation* article in a report are also independently more likely to be using research instrumentally in multiple outputs. Qualitative follow up research could provide greater detail to deepen understandings of examples of instrumental use of *The Conversation* articles.

Working as a politician, policy officer, or government employee also predicted use of *The Conversation* articles to inform the development of strategy, policy, programs, etc., i.e. instrumental use. This is an important finding that demonstrates that politicians and policy officers are actively seeking out research evidence and academic expertise on *The Conversation* and using it to inform policy and program development. No other employment type, out of a total of 16 employment types, significantly predicted direct, instrumental use of research. This study shows that publishing in *The Conversation* offers a unique opportunity to directly inform and influence politicians and policy officers, with real potential for that research to be used in government policy and program decision-making.

‘Discuss with friends and colleagues’ is an engagement action that significantly predicted use of *The Conversation* articles to support existing strategy, policy and other work-related decisions. ‘To assist with work’ was the only response from the survey question ‘what are the main reasons you read *The Conversation*’ that predicted instrumental use of a *The Conversation* article. Together, these results highlight that people are accessing *The Conversation* for work-related purposes and further strengthens the assertion that participants’ find *The Conversation* articles content relevant to their work-related decision-making needs and actionable in their context [58,63,65]. The results strongly suggest that *The Conversation* model is an effective means for both providing access to research, as well as communicating research in a way that enables use of research, a necessary precursor to achieving research impact.

Republishing an article significantly predicted instrumental use of *The Conversation* articles. *The Conversation* articles are made freely available using a Creative Commons license, which allows republishing in full at no cost. The finding suggests that participants who republish articles work in roles that have a research or news-related reporting and/or communication and engagement aspect to their work and are using *The Conversation* as a key source of content. One of the main venues that republish *The Conversation* is the Australian Broadcasting Corporation (Australia’s national broadcaster). Article republishing significantly increases the potential for engagement and use beyond *The Conversation* direct readership, therefore also increasing the likelihood of use of *The Conversation* articles leading to positive impact.

Undertaking further research was another engagement action that predicted instrumental use of *The Conversation* articles. This suggests that these participants were seeking out information for the purpose of directly informing understanding or decision-making. It also suggests that *The Conversation* is just one of a number of resources that participants were turning to to inform development of policy, program, strategy, etc. This supports previous research highlighting that research evidence is only ever one of the many inputs that inform policy, program and practice decision-making [27,66–68]. It may also suggest that participants sought to read the underlying research studies often linked to in *The Conversation* articles, however this is not clear from the survey results. The term ‘research’ can be interpreted in myriad ways and is not always used in reference to academic research literature [69]. Deeper understanding of the motivations of these particular participants could illuminate the types of situations and drivers that are motivating their search for research evidence and uncover the other sources of evidence they rely on.

#### Tactical use of The Conversation articles

‘Used the article in a report’ also predicted use of articles to support a work-related decision or action that had already been made. ‘Used article in a report’ was again the strongest predictor for this type of use based on the classification tree. ‘Symbolic’ or ‘tactical’ use of research are terms coined to describe when research is used to support a decision or action made prior to research evidence being sought or examined [13,15,25].

Being employed as a ‘politician, policy officer or government employee’ significantly predicted tactical use of a *The Conversation* article, supporting the claim that politicians only seek evidence to support their agenda. It is often quipped that politicians only seek information that confirms a predetermined stance or interest, sometimes referred to as ‘policy based evidence’, rather than ‘evidence based policy’, and certainly there are situations where this has occurred [70,71]. However, it has been effectively argued that conceptual and tactical use of research at any stage of the policy decision-making process is a valid use of research. Further, there are often occasions where political staffers and policy officers are not in control of the decision-making process [16,72]. In most cases policy decision-makers, at all levels, are responsible for implementation of the political decision-making of the government of the ruling party [16]. As such, indicating this type of tactical use may be related to use of *The Conversation* articles to inform planning for implementation of policy, program or strategy decisions that have been made and/or announced [67]. Focusing on different types of research use and recognising that research is only ever one of a multitude of inputs that inform decision-making reflects the complex reality of policy development; which is not a linear, rational process and which is affected by a broad range of interconnected factors [13,25,69,71,73–76].

That policy decision-makers are using *The Conversation* articles both instrumentally and tactically suggests articles are being used in relation to particular situational and decision-making needs [13]. Previous studies have also demonstrated that policy-decision makers use research both instrumentally and tactically [10,25,35]. A 2016 systematic review [12] has highlighted the pivotal role that motivation and opportunity play in influencing decision-makers use of research. This suggests a more nuanced understanding of the motivations and opportunities that enable research use, potentially through detailed case studies, is urgently needed [69].

Interestingly however, being employed in senior leadership and management positions, regardless of sector, also predicted this type of tactical use of *The Conversation* articles. And whilst employment position was a significant predictor of research use, employment sector was not. Specific positions in a given context were more important than the context itself in predicting use of *The Conversation* articles for work-related purposes. This finding links to the existing literature regarding the role that context plays in the design of interventions seeking to increase research use by public health decision-makers [30,77]. The findings suggest that regardless of the sector, identifying and strategically including those in senior, authoritative roles in intervention design will be critical. This is supported by systematic review findings that identified authority to make decisions and engaging opinion leaders and champions as a key enablers of research use [40,63,69].

Finding that those at the most senior levels in organisations across all industries and politicians and other government decision-makers are using *The Conversation* articles to inform and support work-related decision-making is significant. These results highlight that while partner-based research funding, as evidence of engagement with industry and government, is an important indicator of future potential impact; indirect engagement efforts targeting those in senior-level and government positions also has potential for real impact [59].

#### Conceptual use of *The Conversation* articles

‘To assist with work’ was the only factor that predicted use of *The Conversation* articles to inform work-related discussion, debate and understanding across both the logistic regression and classification tree analyses. This type of use, where research informs understanding without specific action, is defined as conceptual use [13]. Measuring conceptual use via survey is valuable for various reasons. It is similar to social media analysis that can identify if a particular topic or article of content has been discussed in the public sphere. However, *The Conversation* survey data can complement social media analytics as it can capture conceptual use that may only take place in personal, face-to-face conversation [53]. Further, this type of use, as asked about in *The Conversation* survey, is directly focused on whether research has informed research work-related discussion and debate.

Whilst there is no guarantee that conceptual use will shift to an applied type of use, such as tactical or instrumental, it can enable decision-makers to use research to support their business activity when the opportunity becomes available. Through his in-depth research on policy decision-making and development processes Kingdon [72] highlighted that significant policy change and development can occur when the right ‘window of opportunity’ is open. In this way, conceptual use of research and academic expertise as measured in *The Conversation* survey could be considered as an indicator of research moving along the pathway to impact. Measuring conceptual use of research via survey can augment social media analytics, as it is also able to capture instances of private discussion and debate, which cannot be measured easily on a large scale by other means.

#### Use of *The Conversation* to inform one’s own personal attitude and behaviour chnage

Interestingly, discussing articles with friends and colleagues was the only engagement action that predicted use of *The Conversation* to inform personal attitude or behaviour change. Together, the findings regarding discussing *The Conversation* articles with friends and colleagues suggest that this is a very active type of engagement, where one may be seeking to gather opinions to inform their own decision-making or to use the content to influence the opinions or decision-making of others. The finding supports previous studies that have identified discussion and debate as important in enabling research take up [59,78]. Indeed leading research translation focused organisations have established forums to enable deliberative dialogue around research. Many systematic reviews have identified increased interaction, communication and face-to-face engagement as key enablers of research use [11,12,63]. However, the small numbers of interventions that have sought to use this approach show limited evidence of effectiveness [12].

Reading *The Conversation* ‘to explain the news’ significantly predicted use of a *The Conversation* article to inform personal attitude and behaviour change. This finding suggests that for these participants *The Conversation* is providing greater detail and information that helps them understand a particular current issue and inform decision-making. In these instances *The Conversation* is enabling the use of research and academic expertise in ways that can have very direct and significant impact on people and communities. This opportunity for researchers to have a direct impact on the general public is unique. It is estimated that just 25–50% of all academic research is currently free to access on the Internet via open access journals and articles and institutional repositories [79,80]. Even then much of this research remains inaccessible to the general public, as it written for an expert peer academic audience [65]. As such, *The Conversation* as a not-for-profit organisation that translates research evidence and academic expertise for non-academic audiences, fills a critical gap; enabling individuals to make evidence-informed decisions that can shape the direction and outcomes of human experience, actions and worldview through translation and open communication of academic research and expertise.

Participants who indicated ‘expert opinion and facts’ as a main reason for reading *The Conversation* were also predicted to use *The Conversation* articles to inform personal attitude and behaviour change. Similarly ‘to explain the news’, finding that ‘expert opinion and facts’ significantly predicted use of *The Conversation* article suggests that *The Conversation* is providing a reliable source of academic expertise and research evidence that is relevant to their needs and interests [27,69]. In 2015 Alperin [48] completed one of the first comprehensive studies of non-academic use of institutional repositories in South America [46]. Alperin [48] found that those working outside academia represented between 16–25% of users. Of those, between 7.9 and 10.5% were using articles from repositories for personal reasons. Together these results indicate that there is significant capacity to engage with and use of research amongst the general public, and therefore significant potential for research to have impact through open access research communications platforms [47].

#### Limitations

This study is based on a survey that was designed by marketing and communications staff within *The Conversation*. Only the question on use of *The Conversation* was crafted by an academic and based on academic literature. *The Conversation* articles are based on research evidence and also on academic expertise and opinion and as the survey responses are not linked to individual outputs we do not what articles were actually used. This is an issue with much research in the field that has surveyed government decision-makers about their use of research [37]. Despite the limitations this data set has produced findings reflected in the academic literature as highlighted throughout the discussion. The strength of this analysis is that the types of use of *The Conversation* were clearly defined, which has also been an issue in past studies [11,69]. A further limitation is this is based on an Australian survey and therefore the responses reflect use of *The Conversation* by Australians only. However *The Conversation* is also available in the UK, which has also introduced Research Impact assessment in their national research performance frameworks and *The Conversation* is also available in the US, France, Canada, Indonesia and Africa. Future research on use of *The Conversation* in other countries to provide a comparison to the findings would be valuable.

## Conclusions

*The Conversation* annual survey has provided valuable data demonstrating extensive engagement and use of research and academic expertise by those working in sectors beyond academia. Overall, the major contribution of the study are the findings demonstrating that the majority of factors that predicted the four types of use of *The Conversation* articles were related to engagement actions undertaken after reading a *The Conversation* article, participants’ main reasons for reading *The Conversation* and senior and policy-related employment positions. Importantly however, different factors predicted different types of use, highlighting that interventions seeking to achieve research impact through increased engagement, must be based on a more detailed and nuanced understanding of engagement and use that is currently depicted in the rhetoric surrounding ‘research impact’. These findings suggest that senior and influential decision-makers can play a critical role in development of strategies and interventions seeking to support or increase use of research. Further, this study has shown that *The Conversation* is providing research-informed articles that are being used by industry and government decision-makers to inform work-related decision-making and discussion and debate. This indicates that decision-makers are finding content on *The Conversation* that is relevant to their decision-making needs and actionable in their context. This highlights that, for academics, publishing in *The Conversation* provides an excellent opportunity to increase research use, and therefore potential research impact.

## Supporting information

S1 File Survey questions. (DOCX)

S2 File Total predictors list. (DOCX)

S3 File Regression results. (DOCX)
