# Supplementary material for: Does engagement predict research use? An analysis of The Conversation Annual Survey 2016
Source: PLoS One. 2018 Feb 7;13(2):e0192290. doi: 10.1371/journal.pone.0192290 (PMC5802909; doi:10.1371/journal.pone.0192290)
Supplement: S1 File — (DOCX) [file pone.0192290.s001.docx]

# Attachment 1.

## *The Conversation* Annual Survey Questions and Response Options 2016.

| Q1 If you consent to participate, please click “yes”.  Yes  No |
| --- |
| Q2 What are the main reasons you read The Conversation? (Select all that apply)  To explain the news  To assist in my work / research  To explore issues I care about / for interest  For expert opinions and facts  To assist me in my study  To read about issues not covered elsewhere  To find out about new research and breakthroughs  It is better than the alternatives  Other (please specify) |
| Q3 How frequently do you read our articles?  More than once a day  Daily  A few times a week  Weekly  Monthly  Infrequently  Whenever I need to know about a specific topic |
| Q4 How likely are you to recommend The Conversation to others?  Extremely unlikely =0, 1, 2, 3, 4, 5, 6, 7, 8, 9, Extremely likely = 10 |
| Q5 How much do you value these aspects of The Conversation?  Academic expertise  Research findings  Clarity of writing  Editorial independence  No commercial agenda  Design and usability  Creative commons / open source publishing  Author disclosures  Variety of topics covered  Opportunity to engage with people outside my normal  networks |
| Q6 What actions have you taken as a result of reading an article on The Conversation? (please select all that apply)  Republished the article  Left a comment on the article  Shared an article on social networks (e.g. Facebook, Twitter) or by email  Discussed with friends or colleagues  Printed to read or share  Contacted the author to discuss their ideas  Contacted the author to work with them  Contacted the author to ask about studying with them or at their university  Used the article in a report  Used the article as a classroom resource or as basis of discussion with students  Contacted a local politician or government official  Undertaken further research  None |
| Q7 Have you used articles from The Conversation to do any of the following? (please select all that apply)  Inform general understanding, discussion and debate on strategy, policy, project or business topics  Develop strategy, policy, presentations, decisions and/or directions which have been documented, for example, in policy briefs, papers, projects plans  or reports, PowerPoints, etc.  Further support existing an strategy, policy, program or business decisions  Change my own behaviour and/or attitudes in my personal life  Did not influence or change anything  Other uses not listed above |
| Q8 Have you published an article with The Conversation in the last 12 months?  Yes  No |
| Q9 Which university or research institute are you affiliated with?(please select all that apply)  Adelaide  ACU  ANU  Bond  Canberra  Central Queensland  Charles Darwin  Charles Sturt  Curtin  Deakin  ECU  Federation  Flinders  Griffith  James Cook  La Trobe  Macquarie  Melbourne  Monash  Murdoch  New England  UNSW Australia  Newcastle  Notre Dame  QUT  RMIT  UniSA  Southern Cross  Southern Queensland  Sunshine Coast  Swinburne  Sydney  Torrens  UQ  UTas  UTS  UWA  Victoria University  Western Sydney  Wollongong |
| Q10 Did publishing your article on The Conversation lead to any of the following? (please select all that apply)  Invitations to speak at conferences  Requests to write or be interviewed by another publication or media outlet  Contact for research collaboration  Discussions with students  Discussions with friends, colleauges, or the general public  Contact by business / industry (e.g. to consult)  None of these  Can you tell us more? |
| Q11 What kind of media follow up?  Newspaper  Radio  Website or blog  Television |
| Q12 Have you used your author dashboard for any of the following?  Tracking public engagement with my articles  Tracking what countries are reading my article  Tracking which publications are republishing my article  Monitoring and responding to comments and social media (e.g. Facebook, Twitter)  Demonstrating engagement as part of a performance evaluation / annual review  Demonstrating engagement to apply for research funding  Demonstrating engagement as part of a job application  None of these  Other (please specify) |
| Q13 How old are you?  Under 18  18-25  26-35  36-49  50-64  65 or older |
| Q14 What is your sex / gender identity?  Male  Female  Transgender  I wish not to say |
| Q15 Where do you live?  VIC  NSW  ACT  QLD  NT  WA  SA  TAS  Outside Australia (please specify country) |
| Q16 What best describes where you live?  Inner city / Suburban  Provincial / Regional  Rural / Remote |
| Q17 What best describes your employment status?  Employed, full time  Employed, part time  Unpaid work / volunteer  Not employed  Retired  Carer  Not able to work |
| Q18 Are you currently studying?  Yes, full time  Yes, part time  Planning to within the next 2 years  No |
| Q19 At what type of institution do you study/ intend to study?  High school  TAFE or vocational college  University  Other |
| Q20 What is your combined pre-tax household income? (optional)  Less than $50,000  $49,999 to $99,999  $100,000 -$149,000  $150,000 -$299,000  $300,000 plus  Prefer not to say |
| Q21 What is your highest level of education?  High school  Vocational education and training  Undergraduate Degree  Graduate/Postgraduate Certificate  Graduate/Postgraduate Diploma  Master's Degree  Doctorate  Prefer not to say |
| Q22 Which university awarded your highest qualification?  Adelaide  ACU  ANU  Bond  Canberra  Central Queensland  Charles Darwin  Charles Sturt  Curtin  Deakin  ECU  Federation  Flinders  Griffith  James Cook  La Trobe  Macquarie  Melbourne  Monash  Murdoch  New England  UNSW Australia  Newcastle  Notre Dame  QUT  RMIT  UniSA  Southern Cross  Southern Queensland  Sunshine Coast  Swinburne  Sydney  Torrens  UQ  UTas  UTS  UWA  Victoria University  Western Sydney  Wollongong |
| Q23 What best describes the sector you work in, or last worked in?  Academia & Research  Banking & Financial Services  Consulting & Strategy  Energy & Resources  Art, Design & Architecture  Engineering  Farming & Primary Production  Government, Policy or Public Sector  Healthcare & Medical  Information Technology (IT)  Marketing, Public Relations & Communications  NGO or Social Venture  Science & Technology  Teaching & Education  Media / Journalism  Other (please specify) |
| Q24 What best describes your current job title, or the last job title you had?  Chairperson, director, CEO/CFO,COO, owner, partner or proprietor  General manager, department head, senior executive, manager, or professional  Politician, policy officer, or government employee  Academic, researcher, or knowledge worker (e.g., librarian)  Project officer/support, assistant, or advisor  Health practitioner or clinician  Media professional (e.g., journalist, writer, broadcaster, advertiser, PR)  Teacher  Not applicable  Other |
| Q25 Does anyone in your household own or manage a business?  Yes  No |
| Q26 What best describes the type of business?  Agriculture, forestry, fishing and hunting  Mining  Manufacturing  Electricity, gas and water supply  Construction  Wholesale trade  Retail trade  Accommodation, cafes and restaurants  Transport and storage  Communication services  Finance and insurance  Property and business services  Government administration and defence  Education  Health and community services  Cultural and recreational services  Personal and other services  Other (please specify) |
| Q27 Are you aware we are a registered charity and accept donations from readers?  Yes  No |
| Q28 Would you consider supporting this project by becoming a "Friend of The Conversation" and donating?  Yes  No  Unsure  I'm already a "Friend' who donates |
| Q29 Do you have any other comments, questions, or concerns? |
| Q30 Please provide your contact details in case we have any follow-up questions. (We won't share this with others, it's just for the survey and it is optional), Finally, please sign up to our daily newsletter, or follow us on Facebook and Twitter, and encourage others to. We don't spend any money on advertising, so you spreading the word helps us grow. Thank you. |
