# Supplementary material for: Does engagement predict research use? An analysis of The Conversation Annual Survey 2016
Source: PLoS One. 2018 Feb 7;13(2):e0192290. doi: 10.1371/journal.pone.0192290 (PMC5802909; doi:10.1371/journal.pone.0192290)
Supplement: S2 File — (DOCX) [file pone.0192290.s002.docx]

# Attachment 2.

## Predictors included in regression analyses.

| Engagement Actions |
| --- |
| Republished the article |
| Left a comment on the article |
| Shared an article on social networks (e.g. Facebook, Twitter) or by email |
| Discussed with friends or colleagues |
| Printed to read or share |
| Contacted the author to discuss their ideas |
| Contacted the author to work with them |
| Contacted the author to ask about studying with them or at their university |
| Used the article in a report |
| Used the article as a classroom resource or as basis of discussion with students |
| Contacted a local politician or government official |
| Undertaken further research |
| None |
| Main Reasons for Reading TC |
| To explain the news |
| To assist in my work / research |
| To explore issues I care about / for interest |
| For expert opinions and facts |
| To assist me in my study |
| To read about issues not covered elsewhere |
| To find out about new research and breakthroughs |
| It is better than the alternatives |
| Other (please specify) |
| Value |
| Academic expertise |
| Research findings |
| Clarity of writing |
| Editorial independence |
| No commercial agenda |
| Design and usability |
| Creative commons / open source publishing |
| Author disclosures |
| Variety of topics covered |
| Opportunity to engage with people outside my normal networks |
| Publication Follow Up |
| Invitations to speak at conferences |
| Requests to write or be interviewed by another publication or media outlet |
| Contact for research collaboration |
| Discussions with students |
| Discussions with friends, colleagues, or the general public |
| Contact by business / industry (e.g. to consult) |
| None of these |
| Can you tell us more? |
| Dashboard Use |
| Tracking public engagement with my articles |
| Tracking what countries are reading my article |
| Tracking which publications are republishing my article |
| Monitoring and responding to comments and social media (e.g. Facebook, Twitter) |
| Demonstrating engagement as part of a performance evaluation / annual review |
| Demonstrating engagement to apply for research funding |
| Demonstrating engagement as part of a job application |
| None of these |
| Other (please specify) |
| Employ Status |
| Employed, full time |
| Employed, part time |
| Unpaid work / volunteer |
| Not employed |
| Retired |
| Carer |
| Not able to work |
| Income |
| Less than $50,000 |
| $49,999 to $99,999 |
| $100,000 -$149,000 |
| $150,000 -$299,000 |
| $300,000 plus |
| Prefer not to say |
| Education |
| High school |
| Vocational education and training |
| Undergraduate Degree |
| Graduate/Postgraduate Certificate |
| Graduate/Postgraduate Diploma |
| Master's Degree |
| Doctorate |
| Prefer not to say |
| Sector |
| Academia & Research |
| Banking & Financial Services |
| Consulting & Strategy |
| Energy & Resources |
| Art, Design & Architecture |
| Engineering |
| Farming & Primary Production |
| Government, Policy or Public Sector |
| Healthcare & Medical |
| Information Technology (IT) |
| Marketing, Public Relations & Communications |
| NGO or Social Venture |
| Science & Technology |
| Teaching & Education |
| Media / Journalism |
| Other (please specify) |
| Role title/Employment Position |
| Chairperson, director, CEO/CFO,COO, owner, partner or proprietor |
| General manager, department head, senior executive, manager, or professional |
| Politician, policyofficer, or government employee |
| Academic, researcher, or knowledge worker (e.g., librarian) |
| Project officer/support, assistant, or advisor |
| Health practitioner or clinician |
| Media professional (e.g., journalist, writer, broadcaster, advertiser, PR) |
| Teacher |
| Not applicable |
| Other |
| Business Type |
| Agriculture, forestry, fishing and hunting |
| Mining |
| Manufacturing |
| Electricity, gas and water supply |
| Construction |
| Wholesale trade |
| Retail trade |
| Accommodation, cafes and restaurants |
| Transport and storage |
| Communication services |
| Finance and insurance |
| Property and business services |
| Government administration and defence |
| Education |
| Health and community services |
| Cultural and recreational services |
| Personal and other services |
| Other (please specify) |
| Age |
| Under 18 |
| 18-25 |
| 26-35 |
| 36-49 |
| 50-64 |
| 65 or older |
