# Supplementary material for: Does engagement predict research use? An analysis of The Conversation Annual Survey 2016
Source: PLoS One. 2018 Feb 7;13(2):e0192290. doi: 10.1371/journal.pone.0192290 (PMC5802909; doi:10.1371/journal.pone.0192290)
Supplement: S3 File — (DOCX) [file pone.0192290.s003.docx]

# Attachment 3. Regression Results.

# Logistic Regression Tables

**Table 1. Predictors of use of TC to inform work related decision-making.**

|  | | | | | | | | | |
| --- | --- | --- | --- | --- | --- | --- | --- | --- | --- |
|  | | B | S.E. | Wald | df | Sig. | Exp(B) | 95% C.I.for EXP(B) | |
|  |  |  |  |  |  |  |  | Lower | Upper |
|  | Main Reason_Work | .909 | .085 | 113.809 | 1 | .000 | 2.481 | 2.100 | 2.932 |
|  | Action_Republish | .660 | .094 | 48.908 | 1 | .000 | 1.934 | 1.608 | 2.327 |
|  | Action_Comment | .254 | .091 | 7.774 | 1 | .005 | 1.289 | 1.078 | 1.540 |
|  | Action_Share | .225 | .085 | 6.972 | 1 | .008 | 1.253 | 1.060 | 1.481 |
|  | Action_Print | .423 | .079 | 28.478 | 1 | .000 | 1.526 | 1.307 | 1.782 |
|  | Action_Report | 1.457 | .085 | 290.851 | 1 | .000 | 4.294 | 3.632 | 5.077 |
|  | Action_Teach | .424 | .109 | 15.242 | 1 | .000 | 1.528 | 1.235 | 1.891 |
|  | Action_Contact Goverment | .538 | .121 | 19.713 | 1 | .000 | 1.712 | 1.350 | 2.171 |
|  | Action_Further Research | .611 | .080 | 57.763 | 1 | .000 | 1.842 | 1.573 | 2.156 |
|  | Publication Followup _Invitations | 1.568 | .405 | 14.954 | 1 | .000 | 4.795 | 2.166 | 10.613 |
|  | Dashboard Use_Other | 1.982 | .865 | 5.247 | 1 | .022 | 7.254 | 1.331 | 39.530 |
|  | Employment Status_Retired | -.369 | .114 | 10.450 | 1 | .001 | .691 | .553 | .865 |
|  | Income($100,000-$149,000) | .386 | .147 | 6.880 | 1 | .009 | 1.471 | 1.103 | 1.964 |
|  | Income($150,000-$299,000) | .579 | .148 | 15.206 | 1 | .000 | 1.784 | 1.334 | 2.387 |
|  | Income ($300,000 plus) | .848 | .203 | 17.481 | 1 | .000 | 2.334 | 1.569 | 3.474 |
|  | Education_Masters&Doctorate | .570 | .152 | 14.040 | 1 | .000 | 1.769 | 1.313 | 2.384 |
|  | Role Title_Chairperson, Director, etc. | .601 | .180 | 11.118 | 1 | .001 | 1.824 | 1.281 | 2.596 |
|  | Role Title_General manager, department head, senior executive, etc. | .535 | .164 | 10.696 | 1 | .001 | 1.708 | 1.239 | 2.354 |
|  | Role Title_Politician, policy officer, or government employee | 1.169 | .183 | 40.662 | 1 | .000 | 3.219 | 2.247 | 4.611 |
|  | Business Type_ Accommodation, cafes and restaurants | -1.310 | .562 | 5.431 | 1 | .020 | .270 | .090 | .812 |
|  | Business Type_Transport and storage | 1.425 | .577 | 6.099 | 1 | .014 | 4.159 | 1.342 | 12.891 |
|  | Constant | -4.057 | .254 | 254.958 | 1 | .000 | .017 |  |  |

The final model was statistically significant (*χ* ^2^ (48, *N* = 7772) = 1565.023, *p=* <0.001), indicating that the model was somewhat able to distinguish between participants who did and did not use research.

The model overall explained between 19.7% (Cox and Snell R square) and 33.9% (Nagelkerke R square) of the variance in research use and correctly classified 86.8% of cases. The number of positive cases of use of evidence (*N* =1139) is also appropriate for the model, in that there are at least ten positive cases per variable.

The table below shows the factors in the model all had a variance inflation factor (VIF) less than 2, indicating that there was no issue with multi-co-linearity.

**Table 1a. Collinearity results for predictors of use of TC to inform work related decision-making.**

| Model | | Collinearity Statistics | |
| --- | --- | --- | --- |
|  |  | Tolerance | VIF |
| 1 | (Constant) |  |  |
|  | Main Reason_Work | .769 | 1.300 |
|  | Action_Republish | .935 | 1.069 |
|  | Action_Share | .941 | 1.063 |
|  | Action_Print | .919 | 1.088 |
|  | Action_Report | .854 | 1.171 |
|  | Action_Teach | .893 | 1.120 |
|  | Action_Contact Government | .958 | 1.044 |
|  | Action_Further Research | .924 | 1.082 |
|  | PubFollowup_Invitations | .967 | 1.034 |
|  | Dashboard Use_other_quant | .975 | 1.025 |
|  | Employment Status_Retired | .827 | 1.209 |
|  | Income | .908 | 1.101 |
|  | Education | .949 | 1.054 |
|  | Role Title | .945 | 1.058 |
|  | Business Type | .953 | 1.049 |

**Table 2. Predictors of use of TC to support work-related decisions previously made.**

|  | | | | | | | | | |
| --- | --- | --- | --- | --- | --- | --- | --- | --- | --- |
|  | | B | S.E. | Wald | df | Sig. | Exp(B) | 95% C.I.for EXP(B) | |
|  |  |  |  |  |  |  |  | Lower | Upper |
|  | Action_Republish | .575 | .088 | 42.889 | 1 | .000 | 1.777 | 1.496 | 2.110 |
|  | Action_Share | .242 | .076 | 10.025 | 1 | .002 | 1.274 | 1.097 | 1.479 |
|  | Action_Discuss | .482 | .121 | 15.895 | 1 | .000 | 1.620 | 1.278 | 2.053 |
|  | Action_Print | .416 | .071 | 33.864 | 1 | .000 | 1.515 | 1.317 | 1.743 |
|  | Action_Author Discuss | .401 | .119 | 11.311 | 1 | .001 | 1.493 | 1.182 | 1.886 |
|  | Action_Report | 1.075 | .082 | 172.036 | 1 | .000 | 2.930 | 2.495 | 3.440 |
|  | Action_Teach | .209 | .105 | 3.985 | 1 | .046 | 1.233 | 1.004 | 1.514 |
|  | Action_Contact Goverment | .602 | .105 | 32.863 | 1 | .000 | 1.826 | 1.486 | 2.243 |
|  | Action_Further Research | .547 | .073 | 56.364 | 1 | .000 | 1.729 | 1.499 | 1.994 |
|  | Dashboard Use_Funding | .926 | .409 | 5.125 | 1 | .024 | 2.524 | 1.132 | 5.627 |
|  | Employment Status_FT | .578 | .080 | 52.380 | 1 | .000 | 1.783 | 1.525 | 2.086 |
|  | Employment Status_Unpaid | .396 | .143 | 7.711 | 1 | .005 | 1.486 | 1.124 | 1.966 |
|  | Income ($49,999-$99,999) | .329 | .128 | 6.623 | 1 | .010 | 1.390 | 1.082 | 1.786 |
|  | Income ($100,000-$149,000) | .485 | .135 | 12.890 | 1 | .000 | 1.624 | 1.246 | 2.115 |
|  | Income ($150,000-$299,000) | .443 | .139 | 10.220 | 1 | .001 | 1.558 | 1.187 | 2.045 |
|  | Income ($300,000 plus) | .409 | .193 | 4.476 | 1 | .034 | 1.505 | 1.031 | 2.198 |
|  | Sector_Consulting & Strategy | .706 | .176 | 15.996 | 1 | .000 | 2.025 | 1.433 | 2.862 |
|  | Sector_Energy & Resources | .484 | .242 | 4.001 | 1 | .045 | 1.622 | 1.010 | 2.607 |
|  | Sector_Government, Policy or Public Sector | .353 | .148 | 5.701 | 1 | .017 | 1.424 | 1.065 | 1.902 |
|  | Sector_Media / Journalism | -1.423 | .407 | 12.224 | 1 | .000 | .241 | .109 | .535 |
|  | Role Title_Chairperson, Director, etc. | .646 | .152 | 18.190 | 1 | .000 | 1.909 | 1.418 | 2.569 |
|  | Role Title_General manager, department head, senior executive, etc. | .388 | .140 | 7.654 | 1 | .006 | 1.474 | 1.120 | 1.940 |
|  | Role Title_Politician, policy officer, or government employee | .454 | .180 | 6.360 | 1 | .012 | 1.574 | 1.106 | 2.239 |
|  | Value_Engage | .099 | .028 | 12.372 | 1 | .000 | 1.104 | 1.045 | 1.167 |
|  | Constant | -4.167 | .222 | 350.763 | 1 | .000 | .016 |  |  |
|  | | | | | | | | | |

The final model was statistically significant (*χ* ^2^ (42, *N* =7772) = 997.556, *p=* <0.001), indicating that the model was somewhat able to distinguish between participants who did and did not use research.

The model overall explained between 13.7% (Cox and Snell R square) and 22.2% (Nagelkerke R square) of the variance in research use and correctly classified 82.5% of cases. The number of positive cases of use of evidence (*N* =1326) is also appropriate for the model, in that there are at least ten positive cases per variable.

The factors in the model all had VIFs less than 2, indicating that there was no issue with multi-co-linearity.

**Table 2a. Collinearity results for predictors of use of TC to support work-related decisions previously made.**

| Model | | Collinearity Statistics | |
| --- | --- | --- | --- |
|  |  | Tolerance | VIF |
| 1 | (Constant) |  |  |
|  | Value_Engage | .955 | 1.047 |
|  | Action_Republish | .936 | 1.068 |
|  | Action_Share | .891 | 1.122 |
|  | Action_Discuss | .870 | 1.149 |
|  | Action_Print | .917 | 1.091 |
|  | Action_Author Discuss | .956 | 1.046 |
|  | Action_Report | .895 | 1.117 |
|  | Action_Teach | .918 | 1.089 |
|  | Action_Contact Government | .955 | 1.047 |
|  | Action_Further Research | .931 | 1.074 |
|  | Dashuse_Funding | .982 | 1.019 |
|  | Employment Status_FT | .790 | 1.266 |
|  | Employment Status_Unpaid | .948 | 1.055 |
|  | Income | .866 | 1.154 |
|  | Sector | .933 | 1.072 |
|  | Role Title | .898 | 1.114 |

**Table 3. Predictors of use of TC to inform discussion and debate related to work.**

|  | | | | | | | | | |
| --- | --- | --- | --- | --- | --- | --- | --- | --- | --- |
|  | | B | S.E. | Wald | df | Sig. | Exp(B) | 95% C.I.for EXP(B) | |
|  |  |  |  |  |  |  |  | Lower | Upper |
|  | Main Reason_Explain News | .298 | .052 | 33.408 | 1 | .000 | 1.347 | 1.218 | 1.491 |
|  | Main Reason_Work | .373 | .070 | 28.745 | 1 | .000 | 1.452 | 1.267 | 1.664 |
|  | Main Reason_Issues | .561 | .066 | 72.780 | 1 | .000 | 1.752 | 1.540 | 1.993 |
|  | Action_Republish | .712 | .090 | 62.763 | 1 | .000 | 2.038 | 1.709 | 2.430 |
|  | Action_Author Discuss | .497 | .124 | 16.094 | 1 | .000 | 1.645 | 1.290 | 2.097 |
|  | Action_Report | .904 | .097 | 86.111 | 1 | .000 | 2.470 | 2.041 | 2.990 |
|  | Action_Further Research | .710 | .067 | 111.378 | 1 | .000 | 2.034 | 1.783 | 2.321 |
|  | Employment Status_FT | .648 | .055 | 140.205 | 1 | .000 | 1.912 | 1.717 | 2.128 |
|  | Constant | -.617 | .067 | 83.775 | 1 | .000 | .539 |  |  |

The final model was statistically significant (*χ* ^2^ (8, *N* =7772) = 889.376, *p=* <0.001), indicating that the model was somewhat able to distinguish between participants who did and did not use research.

The model overall explained between 10.8% (Cox and Snell R square) and 15.0% (Nagelkerke R square) of the variance in research use and correctly classified 68.2% of cases. The number of positive cases of use of evidence (*N* =5149) is also appropriate for the model, in that there are at least ten positive cases per variable.

The factors in the model all had VIFs less than 2, indicating that there was no issue with multi-co-linearity.

**Table 3a. Collinearity results for predictors of use of TC to inform discussion and debate related to work.**

| Model | | Collinearity Statistics | |
| --- | --- | --- | --- |
|  |  | Tolerance | VIF |
| 1 | (Constant) |  |  |
|  | Main Reason_Explain News | .975 | 1.025 |
|  | Main Reason_Work | .827 | 1.209 |
|  | Main Reason_Issues | .973 | 1.027 |
|  | Action_Republish | .961 | 1.041 |
|  | Action_Author Discuss | .966 | 1.036 |
|  | Action_Report | .866 | 1.155 |
|  | Action_Further Research | .932 | 1.073 |
|  | Employment Status_FT | .951 | 1.052 |

**Table 4. Predictors of use of TC to inform changing one’s own attitude or behavior in personal life.**

|  | | | | | | | | | |
| --- | --- | --- | --- | --- | --- | --- | --- | --- | --- |
|  | | B | S.E. | Wald | df | Sig. | Exp(B) | 95% C.I.for EXP(B) | |
|  |  |  |  |  |  |  |  | Lower | Upper |
| Step 1^a^ | Main Reason_Explain News | .394 | .056 | 49.446 | 1 | .000 | 1.483 | 1.329 | 1.656 |
|  | Main Reason_Work | -.448 | .067 | 44.297 | 1 | .000 | .639 | .560 | .729 |
|  | Main Reason_Issues | .337 | .077 | 19.229 | 1 | .000 | 1.400 | 1.205 | 1.628 |
|  | Main Reason_Expertise | .382 | .068 | 31.928 | 1 | .000 | 1.466 | 1.284 | 1.674 |
|  | Main Reason_Only Source | .226 | .059 | 14.790 | 1 | .000 | 1.254 | 1.117 | 1.407 |
|  | Main Reason_Research News | .180 | .057 | 9.816 | 1 | .002 | 1.197 | 1.070 | 1.339 |
|  | Main Reason_Best Option | .318 | .057 | 31.620 | 1 | .000 | 1.375 | 1.231 | 1.536 |
|  | Value_Expertise | .129 | .039 | 10.764 | 1 | .001 | 1.138 | 1.053 | 1.229 |
|  | Value_CCorOA | -.102 | .030 | 11.207 | 1 | .001 | .903 | .851 | .959 |
|  | Value_Author Disclosure | .124 | .033 | 13.714 | 1 | .000 | 1.131 | 1.060 | 1.208 |
|  | Action_Discuss | .696 | .080 | 76.424 | 1 | .000 | 2.005 | 1.716 | 2.344 |
|  | Action_Further Research | .350 | .064 | 30.101 | 1 | .000 | 1.419 | 1.252 | 1.607 |
|  | Age_Combined | -.248 | .096 | 6.687 | 1 | .010 | .781 | .647 | .942 |
|  | Age_Combined | -.596 | .089 | 45.178 | 1 | .000 | .551 | .463 | .656 |
|  | Age_Combined | -.828 | .103 | 64.257 | 1 | .000 | .437 | .357 | .535 |
|  | Employment Status_FT | -.267 | .083 | 10.442 | 1 | .001 | .766 | .652 | .900 |
|  | Employment Status_PT | -.175 | .084 | 4.343 | 1 | .037 | .840 | .712 | .990 |
|  | Education_ Graduate Certificate or Diploma | -.208 | .100 | 4.308 | 1 | .038 | .813 | .668 | .989 |
|  | Education_ Undergraduate | -.203 | .099 | 4.213 | 1 | .040 | .817 | .673 | .991 |
|  | Education_ Masters&Doctorate | -.502 | .096 | 27.087 | 1 | .000 | .605 | .501 | .731 |
|  | Role Title_ Chairperson, Director, etc. | -.291 | .121 | 5.824 | 1 | .016 | .748 | .590 | .947 |
|  | Role Title_ Media professional | -.368 | .171 | 4.638 | 1 | .031 | .692 | .495 | .967 |
|  | Role Title­_ Not applicable | -.344 | .129 | 7.072 | 1 | .008 | .709 | .550 | .913 |
|  | Business Type_ Construction | .883 | .303 | 8.487 | 1 | .004 | 2.418 | 1.335 | 4.379 |
|  | Business Type_ Transport and storage | 1.371 | .593 | 5.339 | 1 | .021 | 3.940 | 1.231 | 12.609 |
|  | Business Type_ Finance and insurance | .652 | .319 | 4.188 | 1 | .041 | 1.920 | 1.028 | 3.586 |
|  | Business Type_ Health and community services | .590 | .181 | 10.580 | 1 | .001 | 1.804 | 1.264 | 2.574 |
|  | Business Type_ Cultural and recreational services | .500 | .234 | 4.554 | 1 | .033 | 1.648 | 1.042 | 2.608 |
|  | Constant | -1.516 | .358 | 17.899 | 1 | .000 | .220 |  |  |
|  | | | | | | | | | |

The final model was statistically significant (*χ* ^2^ (59, *N* =7772) = 1026.435, *p=* <0.001), indicating that the model was somewhat able to distinguish between participants who did and did not use research.

The model overall explained between 14.1% (Cox and Snell R square) and 18.9% (Nagelkerke R square) of the variance in research use and correctly classified 66.7% of cases. The number of positive cases of use of evidence (*N* =4112) is also appropriate for the model, in that there are at least ten positive cases per variable.

The factors in the model all had VIFs less than 2, indicating that there was no issue with multi-co-linearity.

**Table 4a. Collinearity results for predictors of use of TC to inform changing one’s own attitude or behavior in personal life.**

| Model | | Collinearity Statistics | |
| --- | --- | --- | --- |
|  |  | Tolerance | VIF |
| 1 | (Constant) |  |  |
|  | MainReason_ExplainNews | .884 | 1.131 |
|  | MainReason_Work | .848 | 1.179 |
|  | MainReason_Issues | .909 | 1.100 |
|  | MainReason_Expertise | .800 | 1.251 |
|  | MainReason_OnlySource | .836 | 1.196 |
|  | MainReason_ResearchNews | .832 | 1.202 |
|  | MainReason_BestOption | .879 | 1.137 |
|  | Value_Expertise | .746 | 1.341 |
|  | Value_CCorOA | .673 | 1.486 |
|  | Value_AuthorDisclosure | .631 | 1.585 |
|  | Action_Discuss | .861 | 1.162 |
|  | Action_FurtherResearch | .907 | 1.102 |
|  | Age_Combined | .702 | 1.424 |
|  | EmployStatus_FT | .561 | 1.784 |
|  | EmployStatus_PT | .701 | 1.427 |
|  | Education_Combined | .941 | 1.063 |
|  | RoleTitle | .948 | 1.055 |
|  | BusinessType | .926 | 1.080 |

## Classification trees

Each node in the tree shows the model group (top), proportion of use (middle) and percent of the sample (bottom). Nodes are coloured from dark blue through to dark green according to the proportion of use.

**Figure 1. Predictors of use of TC to inform work related decision-making.**


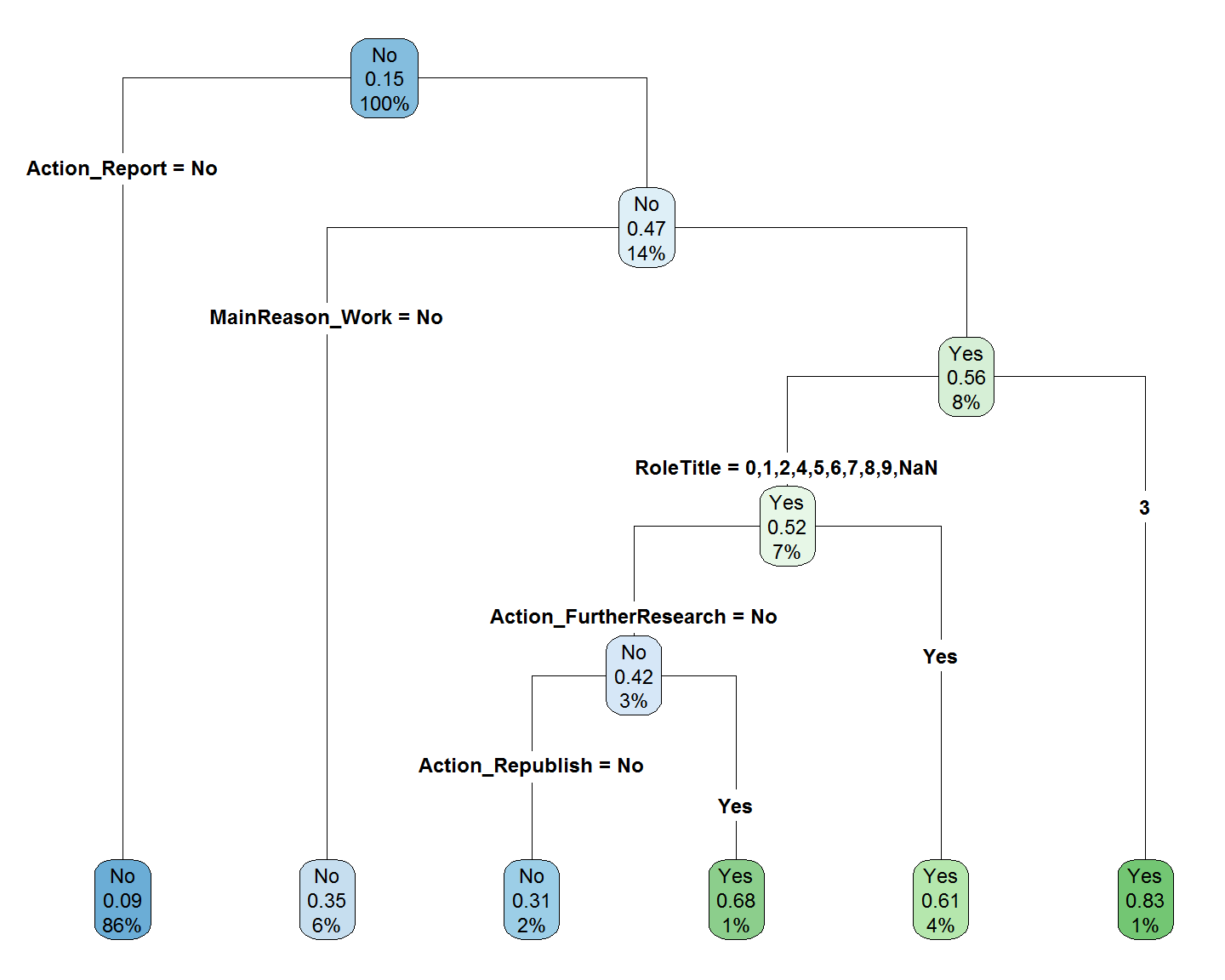


##### **Cross-validated error**


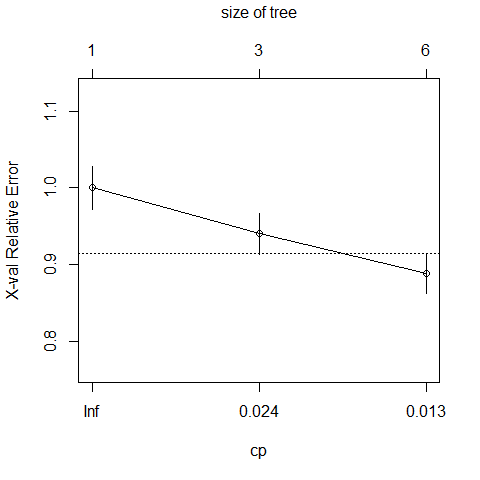


##### **Cross-tabulations of observations and predictions (numbers)**

## Predicted
## No Yes
## No 6474 159
## Yes 836 303

##### **Cross-tabulations of observations and predictions (percents)**

## Predicted
## No Yes
## No 83 2
## Yes 11 4

**Figure 2. Predictors of use of TC to support work-related decisions previously made**


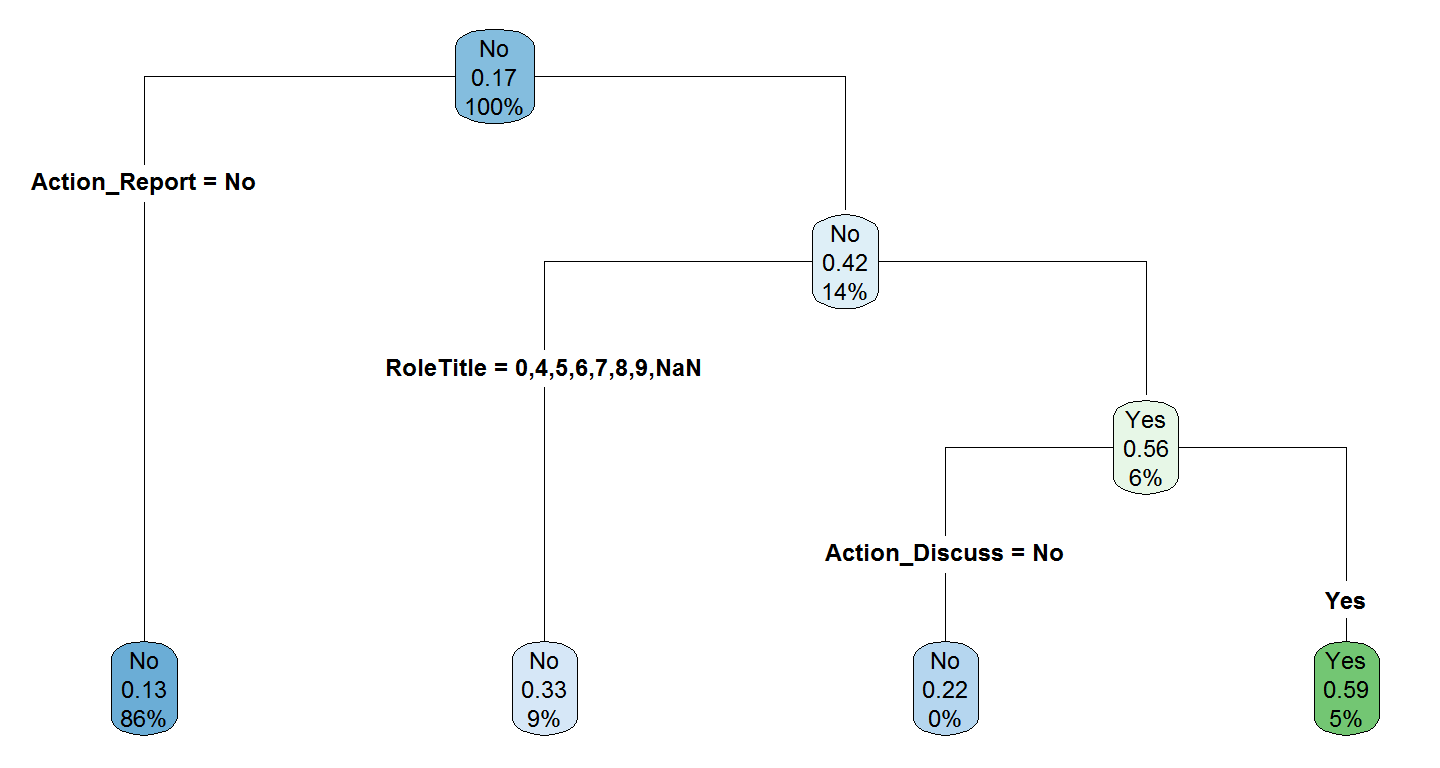


##### **Cross-validated error**


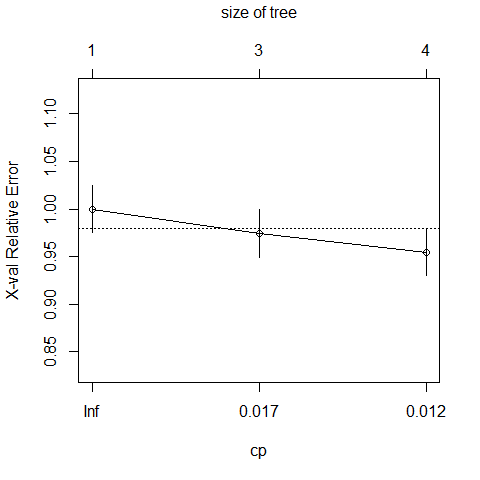


##### **Cross-tabulations of observations and predictions (numbers)**

## Predicted
## No Yes
## No 6285 161
## Yes 1094 232

##### **Cross-tabulations of observations and predictions (percents)**

## Predicted
## No Yes
## No 81 2
## Yes 14 3

**Figure 3. Predictors of use of TC to inform discussion and debate related to work.**


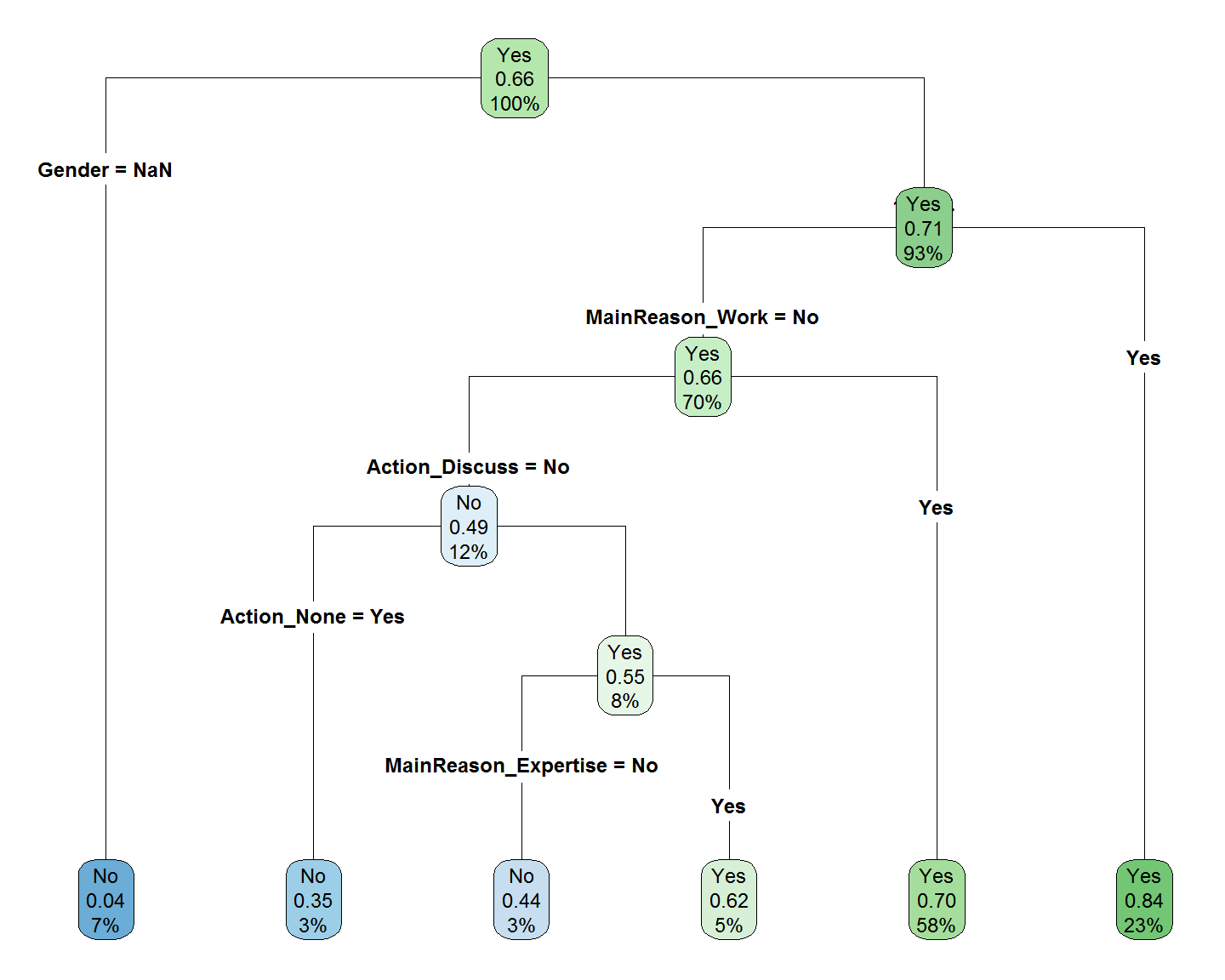


##### **Cross-validated error**


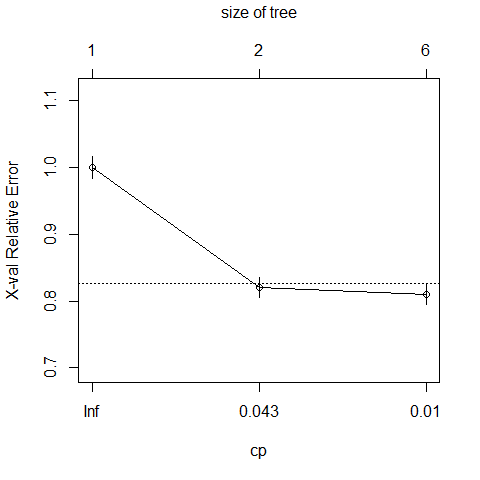


##### **Cross-tabulations of observations and predictions (numbers)**

## Predicted
## No Yes
## No 800 1823
## Yes 221 4928

##### **Cross-tabulations of observations and predictions (percents)**

## Predicted
## No Yes
## No 10 23
## Yes 3 63

**Figure 4. Predictors of use of TC to inform changing one’s own attitude or behavior in personal life.**


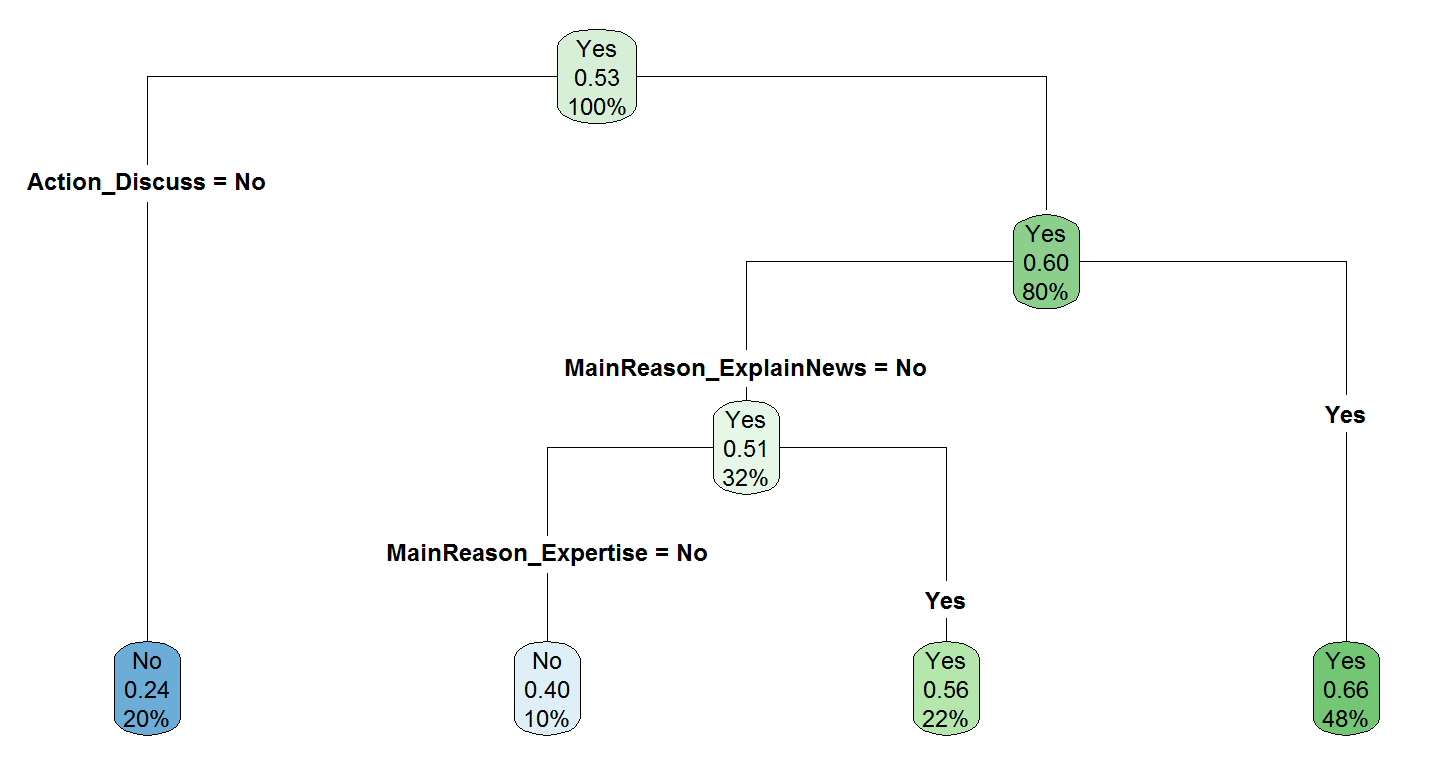


##### **Cross-validated error**


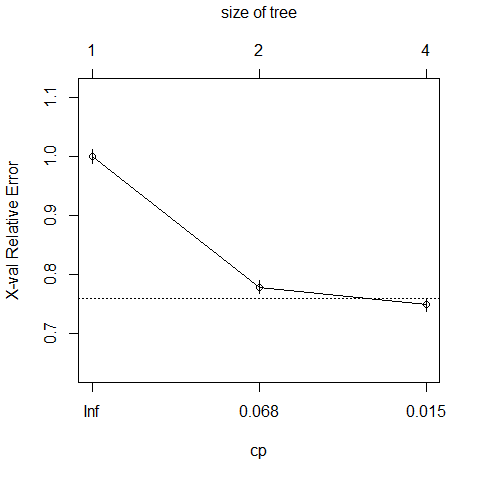


##### **Cross-tabulations of observations and predictions (numbers)**

## Predicted
## No Yes
## No 1672 1988
## Yes 707 3405

##### **Cross-tabulations of observations and predictions (percents)**

## Predicted
## No Yes
## No 22 26
## Yes 9 44
